# Transmembrane Pressure and Recovery of Serum Proteins during Microfiltration of Skimmed Milk Subjected to Different Storage and Treatment Conditions

**DOI:** 10.3390/foods9040390

**Published:** 2020-03-27

**Authors:** Manon Granger-Delacroix, Nadine Leconte, Fabienne Garnier-Lambrouin, Françoise Le Goff, Marieke Van Audenhaege, Geneviève Gésan-Guiziou

**Affiliations:** 1 INRAE, Agrocampus Ouest, STLO, F-35042 Rennes, France; manon.granger-delacroix@inrae.fr (M.G.-D.); nadine.leconte@inrae.fr (N.L.); fabienne.lambrouin@inrae.fr (F.G.-L.); 2 Sodiaal International, Department of Research & Innovation, 75014 Paris, France; francoise.legoff@sodiaal.fr (F.L.G.); marieke.vanaudenhaege@sodiaal.fr (M.V.A.)

**Keywords:** cold storage, heat treatment, 1.4 μm microfiltration, fouling, serum protein

## Abstract

Milk pre-processing steps-storage at 4 °C (with durations of 48, 72 or 96 h) and methods for microbiological stabilization of milk (1.4 μm microfiltration, thermization, thermization + bactofugation, pasteurization) are performed industrially before 0.1 µm-microfiltration (MF) of skimmed milk to ensure the microbiological quality of final fractions. The objective of this study was to better understand the influence of these pre-processing steps and their cumulative effects on MF performances (i.e., transmembrane pressure, and transmission and recovery of serum proteins (SP) in the permeate). Results showed that heat treatment of skimmed milk decreased ceramic MF performances, especially after a long 4 °C storage duration (96 h) of raw milk: when milk was heat treated by pasteurization after 96 h of storage at 4 °C, the transmembrane pressure increased by 25% over a MF run of 330 min with a permeation flux of 75 L·h^−1^·m^−2^ and a volume reduction ratio of 3.0. After 48 h of storage at 4 °C, all other operating conditions being similar, the transmembrane pressure increased by only 6%. When milk was 1.4 µm microfiltered, the transmembrane pressure also increased by only 6%, regardless of the duration of 4 °C storage. The choice of microbiological stabilization method also influenced SP transmission and recovery: the higher the initial heat treatment of milk, the lower the transmission of SP and the lower their recovery in permeate. Moreover, the decline of SP transmission was all the higher that 4 °C storage of raw milk was long. These results were explained by MF membrane fouling, which depends on the load of microorganisms in the skimmed milks to be microfiltered as well as the rate of SP denaturation and/or aggregation resulting from pre-processing steps.

## 1. Introduction

In the dairy industry, crossflow microfiltration (MF) at 50 °C using a membrane with 0.1 μm mean pore size is widely used to separate casein micelles (~150 nm) and serum proteins (SP) (~2–15 nm) [1,2,3]. Retentate, containing mainly casein micelles, has excellent functional properties and is generally used to enrich vat milk for cheese making. Permeate, containing SP, lactose and minerals, is usually ultrafiltered and sometimes demineralized. This results in protein-rich fractions with a high nutritional value dedicated to specific populations such as infants and seniors [4]. The great interest in these fractions explains the increasing number of MF equipment in the dairy industry [5] and the need to better control MF performances and properties of final fractions. To date, studies have examined optimization of operating conditions or impact of milk constituents (casein micelles, soluble proteins and minerals) [6] to explain MF performances but have not considered the thermal history of milk. To capitalize on results of these studies using different milks, the influence of milk history needs to be considered first, especially because in an industrial setting, milk is microfiltered after pre-processing steps that may influence milk components.

Milk is microfiltered industrially at 50 °C after pre-processing steps required to ensure the sanitary quality of milk. Cold storage and microbiological stabilization are thus always performed before MF. More practically, raw milk is first stored at a cold temperature (4–6 °C) for 48–96 h, which corresponds to the duration between the first milk pumped into the collection tank at a farm to the beginning of treatment at the processing plant. At the plant, the milk is skimmed and then either heat treated by pasteurization (72–80 °C for a few seconds) or thermization (<70 °C for a few seconds) with an optional bactofugation (centrifugation) step, or microfiltered at ~50 °C using a 0.8–1.4 µm pore size membrane to reduce the bacteria count. The composition and characteristics of both the retentate and permeate fractions have been observed to depend on the pre-processing methods applied to milk in an industrial setting, but there is no clear understanding of the factors responsible for these differences.

To our knowledge, no study has examined the potential influence of cold storage of raw milk on MF performances and composition of final fractions. During cold storage, some colloidal minerals and caseins are solubilized into the aqueous phase. The distribution of these entities between the colloidal and soluble phases remains stable from 48–96 h of cold storage [7]. However, release of caseins into the soluble phase at low temperature may foster their hydrolysis, which is expected to modify the composition of final fractions by increasing the peptide content in milk. Milk also contains microorganisms, particularly psychrotrophic bacteria, that can proliferate greatly before processing depending on storage conditions [8,9]. The increase in microorganism load during the 48–96 h storage of raw milk could thus decrease MF performances of skimmed milk. Studies of effects of bacterial growth on MF efficacy and quality of milk fractions are lacking.

The influence of heat treatment of milk on MF effectiveness and the properties of fractions has also been rarely studied. In 2010, Hurt and Barbano [10] developed a model to predict SP content in permeate based on a mass balance and the denaturation ratio of SP. They found lower recovery of SP in average permeate after MF of heat-treated milks than after MF of no- or low-heat-treated milks. They assumed that heat treatment decreased only the quantity of proteins that could pass through the membrane (i.e., not denatured SP) but did not consider a possible change in selectivity due to modifications in the deposit layer. To our knowledge, this latter point still needs to be confirmed, and studying MF performances is one way to explore this assumption. In 2018, Zulewska et al. [11] studied the influence of microbiological stabilization of milk (pasteurization, thermization and 1.4 μm microfiltration) on performances of cold (~6 °C) 0.1 µm MF. They found no differences in mean SP transmission or mean decrease in permeation flux among the milks microfiltered. However, they studied cold MF with a volume reduction ratio (VRR) of 1.5, whose conclusions cannot be applied to most current industrial MF plants, in which the operating temperature is maintained at 50 °C and the final VRR exceeds 3.2. In 2014, Svanborg et al. [12] compared MF performances at 50 °C (0.2 μm ceramic membrane, uniform transmembrane pressure (UTP) concept) of pasteurized (73 °C for 15 s) and unpasteurized skimmed milk. They observed lower permeation flux for unpasteurized milk than for pasteurized milk. They also found that the average permeate contained less SP after MF of pasteurized skimmed milk than after MF of unpasteurized milk. It is unclear, however, whether a change in selectivity during MF influenced the results, since only average fractions were quantified. Several potential explanations were discussed but were not sufficient to identify the most likely hypothesis: MF performances could be decreased by denaturation of SP and/or formation of a mineral precipitate (calcium phosphate) caused by heat-treating milk before MF. Thus, long-duration MF in feed-and-bleed mode still needs to be performed to mimic industrial conditions, with a special focus on SP transmission over time to define optimal conditions for separation of milk proteins by MF at 50 °C.

Additionally, cold storage and microbiological stabilization are performed sequentially in the industry. As mentioned, each step modifies milk components and could influence MF performances and the properties of fractions; thus, cumulative effects may occur. Since longer cold storage may increase the bacteria count in milk, the choice of microbiological stabilization method is important: heat treatment should lead to the presence of fragments of bacteria in microbiologically stabilized milk, while 1.4 µm microfiltration should have none. The influence of these bacterial fragments and their amount in the milk to be filtered is unclear, however, and studies are required to understand potential cumulative effects of the pre-processing steps applied to milk before MF. This information is crucial for the dairy industry in order to adapt the microbiological stabilization method used to the cold-storage history of raw milk while producing fractions that meet the expectations of end-users.

The objective of this study was thus to investigate the influence of the duration of cold storage of milk, the method of microbiological stabilization used and their cumulative effect on the performances (i.e., fouling, fraction composition) of skimmed milk MF. To this end, three durations of storage (48, 72 and 96 h at 4 °C) were applied to raw milk and combined with four methods of microbiological stabilization (pasteurization, thermization with or without bactofugation, and 1.4 µm microfiltration) applied to skimmed milk. The pre-processed skimmed milk was then microfiltered under operating conditions as close as possible to industrial practices to ensure that results had a high degree of representativeness: use of ceramic Pall membranes operating in UTP mode [13], regulation of temperature at 50 °C and 330 min of crossflow filtration.

## 2. Materials and Methods

### 2.1. Milks and Pre-Processing Methods

Raw milks (48 h of 4 °C storage) were provided by Entremont Alliance (Bretagne, France) (Table 1). They were stored at 4 °C under continuous stirring in a tank (Dairy Platform, INRAE, UMR1253 Science et Technologie du Lait et de l’Oeuf, Rennes, France) to increase their duration of 4 °C storage to 72 or 96 h. The 4 °C stored milks were then skimmed by centrifugation (50 °C, GEA Westfalia Separator, Château-Thierry, France): the residual fat content of skimmed milks was <0.5 g·kg^−1^, except for one (that had been stored for 48 h) whose fat content was 2.0 g·kg^−1^. After skimming, milks were microbiologically stabilized by four methods:

Microfiltration (M-SMilk) using a 1.4 µm membrane (4.6 m^2^, Pall 1.4, 19P1940, UTP).

Thermization at 68 °C for 20 or 52 s (T-SMilk), sometimes followed by bactofugation.

Pasteurization at 78 °C for 52 s (P-SMilk), which was applied to two milks with different dynamics of bacterial growth during 4 °C storage: “standard bacterial growth” (P1-SMilk)—total viable count (TVC) of bacteria in milk increased from 2.3 × 10^4^ to >5.0 × 10^4^ CFU·mL^−1^ from 48 to 96 h of 4 °C storage, respectively; and “no bacterial growth” (P2-SMilk)—TVC remained stable from 48–96 h at 4 °C: 1.4 × 10^4^ CFU·mL^−1^.

**Figure 1:**
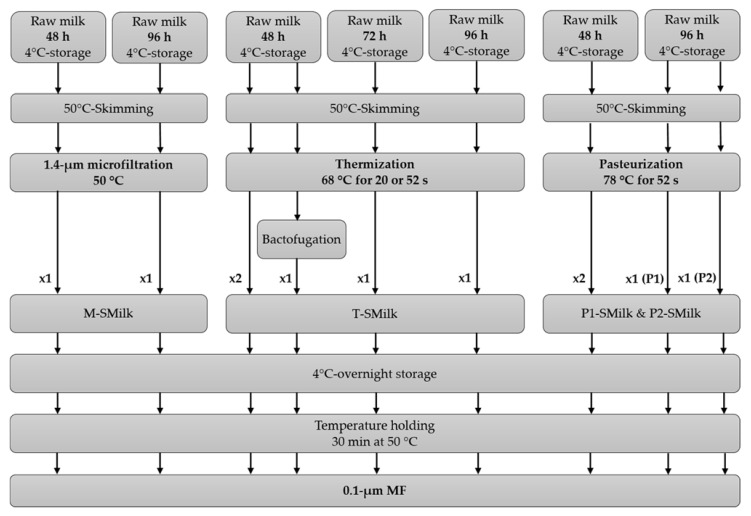
The combinations of 4 °C storage durations and microbiological stabilization methods used before microfiltration (MF) that were studied. Two combinations were performed in duplicate (×2), while the others were performed once (×1). P1-SMilk: pasteurized skimmed milk from raw milk with “standard bacterial growth” during 4 °C storage. P2-SMilk: pasteurized skimmed milk from raw milk with “no bacterial growth” during 4 °C storage.

For practical reasons, microbiologically stabilized milks were stored again at 4 °C overnight before MF. Before MF, skimmed milks were maintained at 50 °C for 30 min to recover a stable mineral balance (Figure 1).

### 2.2. The 0.1-µm MF Setup and Experimental Protocol

A pilot-scale 0.1 μm MF system (Tetra Alcross MFS-7, TetraPak Filtration Systems, Aarhus, Denmark) was equipped with multichannel tubular ceramic membranes (19 channels, inner diameter 4 mm, length 1.02 m, alumina membrane on an alumina support, total membrane area of 1.68 m^2^, 7P1940, Pall, 65, Tarbes, France). MF was performed using the uniform transmembrane pressure (UTP) system, which consists of circulating the permeate at co-current of the retentate to create a pressure drop on the permeate equal to that in the retentate, thus producing no difference in transmembrane pressure (TMP) along the membrane [13,14] and less fouling than a traditional MF system [15].

All experiments were performed with the same cleaned membranes, whose hydraulic resistance (Rm = 3.2 (± 0.1) × 10^11^ m^−1^, calculated according to Darcy’s law) was recovered after cleaning. Before MF, water in the retentate was gently flushed with skimmed milk (corresponding to three times the volume of retentate). During this step, permeate extraction was closed. Water used to rinse the filtration pilot before and after MF came from a network filtered sequentially on 5.0, 1.0 and 0.2 μm cartridges. Each MF run was then divided into two phases:

A concentration phase, which was the same for all experiments, to reach the desired VRR of 3.0. MF was considered to start when the VRR reached 3.0.

A filtration phase of 330 min performed in a feed-and-bleed mode of operation at constant operating parameters: 50 ± 2 °C, VRR of 3.0 ± 0.1, crossflow velocity of 7.0 ± 0.3 m·s^−1^ and permeation flux of 75 ± 1 L·h^−1^·m^−2^.

The change in TMP and transmission of SP, chosen as performance indicators, were assessed during MF. At the end of each experiment, the filtration rig and membranes were rinsed and cleaned with P3 Ultrasil 25F at 1% (*v*/*v*) (alkaline solution, Ecolab, 97, Issy les Moulineaux, France) and nitric acid at 1% (*v*/*v*) (acid solution, HNO_3_; 58% purity, Quaron, Saint-Jacques-de-la-Lande, France). The cleaning was performed at a crossflow velocity of 7 m·s^−1^ in two sequential steps: the 10 first min without permeation and then 10 min with permeation.

Two experiments were performed in duplicate; since they had similar results for the change in TMP (<10% difference between raw data) and transmission of SP (<7% difference between raw data), all other experiments were performed only once (Figure 1).

### 2.3. Analyses

Raw milks stored at 4 °C (48, 72 and 96 h) and skimmed microbiologically stabilized milks entering the MF cartridge were collected for analysis. Retentate and permeate were sampled during MF, and average fractions were collected at the end of MF.

Samples were analyzed for pH and Dornic grade using N/9 NaOH. Fat content was measured by the Gerber method [16]. Dry matter was obtained after desiccation of the sample at 105 °C for 7 h. Ash was measured after combustion of a 5 g sample at 550 °C for 5 h. Total and soluble cation contents (calcium, magnesium) were determined by an atomic absorption spectrophotometer (AA300, Varian France) after dilution of the samples in a solution containing 10% (*v*/*w*) of 6 g·L^−1^ lanthanum chloride and 10% (*v*/*w*) of N/50-hydrochloric acid [17]. Anion content (phosphate, citrate and lactate) was measured by ionic chromatography (Dionex^TM^ ICS 3000, Thermo Fisher Scientific, Les Ulis, France) using guard column (Dionex^TM^ IonPac^TM^ AG 11^TM^, 4 × 50mm, Thermo Fisher Scientific) and analytical column (Dionex^TM^ IonPac^TM^ AS11^TM^, 4 × 250 mm, Thermo Fisher Scientific) coupled with suppressed conductivity detection [18]. Total calcium and magnesium contents were determined from ash solubilized in 1N-hydrochloric acid. Soluble calcium and anions were extracted from samples by ultrafiltration on 10 kDa membranes (Vivaspin VS2002, Sartorius, Göttingen, Germany).

Milk samples were analyzed for TVC, psychrotrophic bacteria count (PBC) and Pseudomonas count. All samples analyzed for bacterial content were prepared and diluted according to the IDF standard (122C, 1996). Samples for TVC determination were incubated for 72 h at 30 °C on plate count agar, as recommended by the IDF (100B, 1991) and described by Piton and Rongvaux-Gaïda (1990) [19]. Samples for PBC determination were incubated for 7 days at 4 °C on plate count agar, as recommended by the IDF (101A, 1991). Samples for Pseudomonas count determination were incubated for 72 h at 25 °C on CFC agar as described by Mead and Adams (1977) [20].

Total nitrogen (TN), non-casein nitrogen (NCN) and non-protein nitrogen (NPN) were determined by the Kjeldahl method according to ISO standard 8968-1 (International Dairy Federation, 2014) [21]. To estimate protein content, TN was multiplied by a conversion factor of 6.38. To estimate protein content in NCN and NPN filtrates, a correction factor was calculated to take into account the weight of precipitate. The experimental error of TN, NCN and NPN was ±1%, ±5% and ±5%, respectively. It is important to note that during precipitation at pH 4.6 for the measurement of NCN, some SP, denatured by previous heat treatments, precipitate with caseins. Conversely, proteose peptones resulting from casein degradation are soluble at pH 4.6. Hence, NCN filtrates from samples contain native SP and proteose peptones.

Contents of α-lactalbumin (α-LA) and β-lactoglobulin (β-LG) were determined in milks, retentates and permeates by reversed phase high-performance liquid chromatography (RP-HPLC) following a protocol adapted from Resmini et al. (1989) [22]. RP-HPLC was performed with a PLRP-S column (PL1912-3801, 300 Å, 8 μm, 150 × 2.1 mm, Agilent Technologies, Thermo Fisher Scientific, Les Ulis, France). Contents of α-LA and β-LG in milks and retentates were determined from the NCN filtrates. In raw milks, the values measured corresponded to the total content of α-LA and β-LG. In microbiologically stabilized milks (M/T/P1/P2-SMilk) and retentates, the values measured corresponded to contents of α-LA and β-LG in the aqueous phase, in either native or partially denatured forms. In permeates, contents of α-LA and β-LG were measured directly in samples, and the values measured corresponded to the total content of α-LA and β-LG. The α-LA and β-LG contents were determined with an experimental error of ± 3%.

The change in TMP during MF (330 min) (ΔTMP) was calculated as follows:

(1) $$\Delta \mathrm{TMP}={\mathrm{TMP}}_{f}\;-\;{\mathrm{TMP}}_{i}$$

with TMP_i_ and TMP_f_ the TMP (10^5^ Pa) at the beginning and end of the 330 min of MF, respectively.

The transmission of SP present in the aqueous phase (Tr) was calculated as follows:

(2) $$\mathrm{Tr}=\frac{C_{p}}{C_{r}}\times 100$$

with C_p_ and C_r_ the content (g·L^−1^) of SP (sum of α-LA and β-LG) in permeate and retentate, respectively, both measured by RP-HPLC.

The recovery ratio of total SP (SPR_k_) was calculated as follows:

(3) $${\mathrm{SPR}}_{k}=\frac{C_{\mathrm{Ap},k}}{C_{\mathrm{SMilk},k}}\times \frac{\mathrm{VRR}-1}{\mathrm{VRR}}$$

with C_Ap,k_ and C_SMilk,k_ the total SP content (g·kg^−1^) in the average permeate and the skimmed milk before microbiological stabilization, respectively, both measured by the Kjeldahl method, and VRR = 3.0.

The recovery ratio of SP present in the aqueous phase (proteins in native or partially denatured forms able to pass through the membrane) (SPR_h_) was calculated as follows:

(4) $${\mathrm{SPR}}_{h}=\frac{C_{\mathrm{Ap},h}}{C_{M/T/P-\mathrm{SMilk},h}}\times \frac{\mathrm{VRR}-1}{\mathrm{VRR}}$$

with C_Ap,h_ and C_M/T/P-SMilk,h_ the content (g·L^−1^) of SP (sum of α-LA and β-LG) in the average permeate and the microbiologically stabilized milk, respectively. Both contents were measured by RP-HPLC.

The denaturation ratio of total SP (DR) was calculated as follows:

(5) $$\mathrm{DR}=\frac{C_{\mathrm{SMilk}}-C_{M/T/P-\mathrm{SMilk}}}{C_{\mathrm{SMilk}}}\times 100$$

where C_SMilk_−C_M/T/P-SMilk_ corresponds to the content of denatured SP (difference between total SP in the aqueous phase before (C_SMilk_) and after (C_M/T/P-SMilk_) microbiological stabilization). Both C_SMilk_ and C_M/T/P-SMilk_ (g·kg^−1^) were measured from NCN filtrates by the Kjeldahl method.

## 3. Results

### 3.1. Composition of Raw Milks after Three 4 °C storage Durations

No large physico-chemical changes in milks were observed from 48–96 h of 4 °C storage (Table 1). Milk pH and Dornic grades did not change, and contents of nitrogen (TN, NCN and NPN), fat, ash, soluble calcium citrate and phosphate were not influenced by 4 °C storage from 48–96 h.

As expected, however, contamination by microorganisms in raw milks increased as the storage duration increased (Table 2). From 48–96 h of 4 °C storage, the TVC in raw milks increased by a factor of ca. 4 (except for the raw milk used to produce P2-SMilk, whose TVC remained ca. 1.5 × 10^4^ CFU·mL^−1^ after 96 h of 4 °C storage). This increase was attributed mainly to the increase in the PBC: from 48–96 h of 4 °C storage, the PBC increased by a factor of 2–50. Notably, the raw milk used to produce P2-SMilk had a much lower PBC (<10 CFU·mL^−1^) after 96 h of storage than P1-SMilk did (>10^4^ CFU·mL^−1^), and its PBC did not change from 48–96 h of 4 °C storage (Table 2).

These results suggest that protein and mineral modifications caused by the decrease in temperature from 37 °C (bovine temperature) to 4 °C had already occurred after 48 h of 4 °C storage. Moreover, they highlight that milk components were not modified or degraded greatly during long-term storage (48–96 h) of raw milk when its initial TVC lay below 10^5^ CFU·mL^−1^. The bacteria content of raw milks increased with the duration of 4 °C storage but did not degrade milk components greatly: the physico-chemical characteristics and composition (pH, Dornic grade, proteins, minerals) of raw milk did not change from 48–96 h of 4 °C storage.

### 3.2. Composition of Skimmed Milks after Microbiological Stabilization

Regardless of the method of microbiological stabilization applied, for a given duration of 4 °C storage, pH, Dornic grade and mineral contents (total calcium, soluble calcium, phosphate and citrate contents) did not differ greatly among M-SMilk (microfiltered at 1.4 µm), T-SMilk (thermized) and P1-SMilk and P2-SMilk (pasteurized) (Table 3). All of the methods effectively rectified the increase in bacteria count caused by 4 °C storage of raw milk: all skimmed milks had a low TVC and a PBC < 10 CFU·mL^−1^, regardless of the storage duration (Table 3). Nonetheless, one can assume that the higher the initial bacteria count of raw milk, the higher the content of cellular debris and non-viable bacteria in heat-treated skimmed milks [23,24]. Thus, P1-SMilk, whose raw milk had the highest bacteria count after 96 h of storage, should have contained more cell fragments than P2-SMilk, whose raw milk had the lowest bacteria count after 96 h. This cellular debris may be retained by the 0.1 µm membrane and then accumulate on the membrane surface during MF.

Heat treatments, which varied in their heat load (time × temperature), modified milk (Table 3). While M-SMilk experienced little denaturation of SP (<1%), as expected given the 50 °C temperature used for 1.4 µm-microfiltration. On the contrary, SP began to denature when skimmed milk was heat-treated, since the pasteurized skimmed milks had a higher percentage of denatured SP (8–13%) than thermized skimmed milks (2–5%) (Table 3). Heat treatment influenced mainly β-LG, which is more prone to aggregate than α-LA: β-LG content in the aqueous phase decreased with heat treatment (e.g., 3.46 vs. 3.26 g·L^−1^ for thermized and pasteurized skimmed milks from raw milk 4 °C stored for 48 h, respectively).

### 3.3. Hydraulic Performances of MF

**Figure 2:**
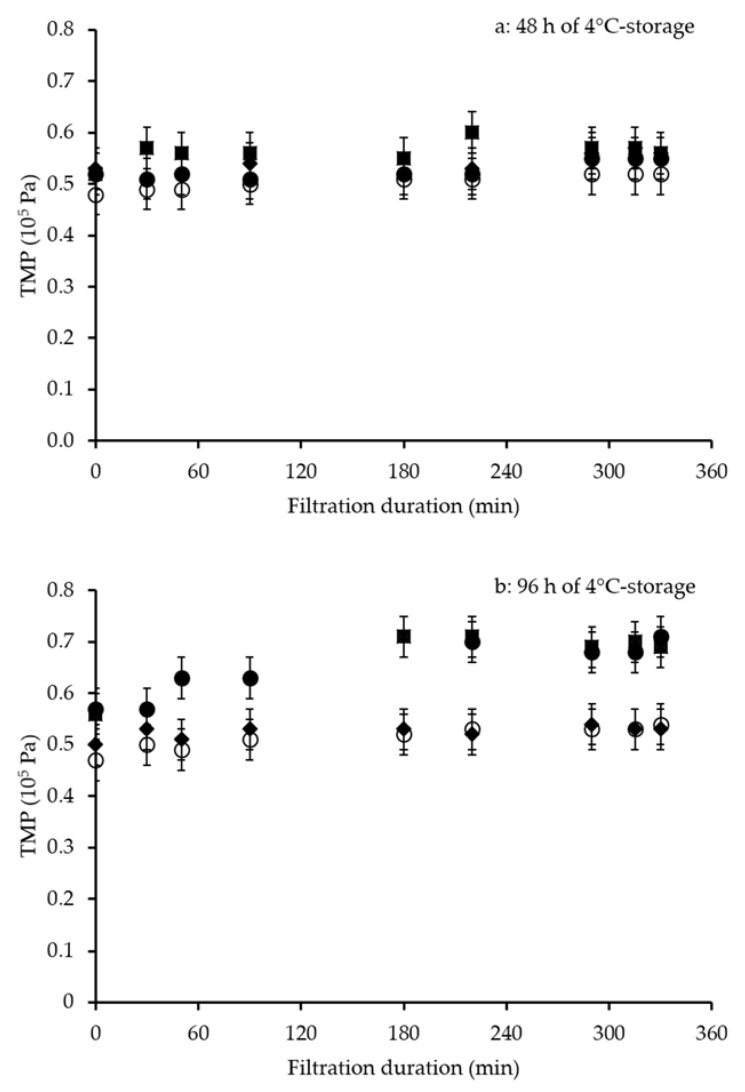
Transmembrane pressure (TMP) during 330 min of microfiltration (MF) of skimmed milk. Milk was microfiltered after (**a**) 48 h or (**b**) 96 h of 4 °C storage of raw milk and different microbiological stabilization methods (diamonds, 1.4 µm microfiltration for M-SMilk; squares, thermization for T-SMilk; solid and open circles, pasteurization for P1-SMilk and P2-SMilk, respectively). Error bars represent 1 experimental error value. Operating conditions: 0.1 μm ceramic membrane; uniform transmembrane pressure system; permeation flux, Jp = 75 L·h^−1^·m^−2^; volume reduction ratio = 3.0; T = 50 °C).

Regardless of the 4 °C storage durations of the initial raw milks and the microbiological stabilization method applied to them, the hydraulic performances of MF were satisfactory: TMP ranged from 0.47–0.71 × 10^5^ Pa during the 330 min MF, indicating little increase in fouling (Figure 2).

To simplify interpretation of results, ∆TMP was used to quantify the fouling that occurred during the MF (Table 4).

### 3.4. Transmission of SP during MF

**Figure 3:**
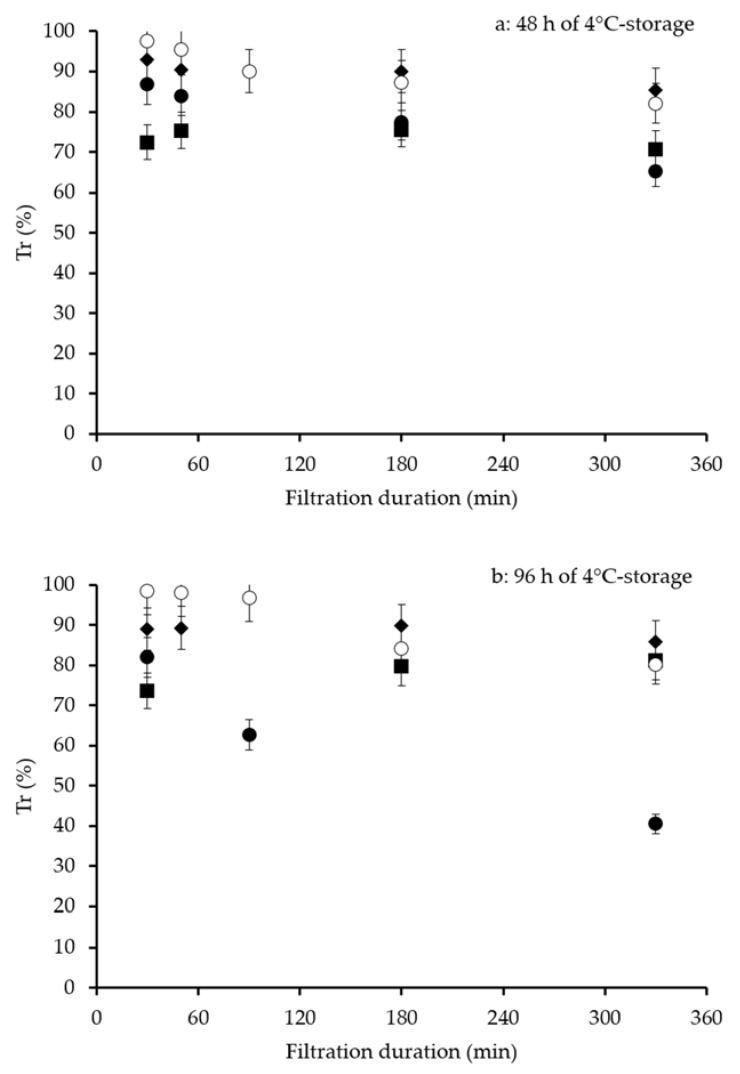
Transmission of serum proteins (α-LA + β-LG), Tr, during skimmed milk filtration. Milk was microfiltered after (**a**) 48 h or (**b**) 96 h of 4 °C storage of raw milk and different microbiological stabilization methods (diamonds, 1.4 µm microfiltration for M-SMilk; squares, thermization for T-SMilk; solid and open circles, pasteurization for P1-SMilk and P2-SMilk, respectively). Error bars represent 1 experimental error value. Operating conditions: 0.1 μm ceramic membrane; uniform transmembrane pressure system; permeation flux, Jp = 75 L·h^−1^·m^−2^; volume reduction ratio = 3.0; T = 50 °C).

For both M-SMilk and T-SMilk, Tr changed little during MF, regardless of the 4 °C storage duration of raw milk (Figure 3). For M-SMilk, Tr remained relatively constant at 90% and 88% for 4 °C storage of 48 and 96 h, respectively. For T-SMilk, Tr ranged from 74% to 78% for 4 °C storage of 48 and 96 h, respectively. Pasteurized milks (P-SMilk) showed two dynamics. For P2-SMilk (“no bacterial growth”), Tr decreased moderately after 330 min of MF (from 98% to 82% for 4 °C storage of 48 h and from 98% to 80% for 4 °C storage of 96 h). Conversely, for P1-SMilk (“standard bacterial growth”), Tr decreased more sharply, from 87% to 65% for 4 °C storage of 48 h and from 82% to 41% for 4 °C storage of 96 h.

### 3.5. Recovery Ratios of SP in Permeate (SPR_k_ and SPR_h_)

For M-SMilk, SPR_k_ were similar for 4 °C storage of 48 and 96 h (56% and 54%, respectively) (Table 5). For T-SMilk, SPR_k_ were also similar for 4 °C storage of 48 and 96 h (50% and 55% respectively). Pasteurized skimmed milks showed two dynamics. For P2-SMilk (“no bacterial growth”), SPR_k_ were nearly the same for 4 °C storage of 48 and 96 h (50% and 51%, respectively). Conversely, for P1-SMilk (“standard bacterial growth”), SPR_k_ decreased from 48% to 41% when 4 °C storage increased from 48 and 96 h.

These results were related to the change in Tr during MF: higher Tr throughout MF resulted in greater recovery of SP. Higher Tr at the beginning of MF did not always result in higher SPR_k_, however, since a decrease in Tr during MF could result in lower SPR_k_ (e.g., P2-SMilk).

SPR_h_ followed the same trend as SPR_k_. For M-SMilk, SPR_h_ were similar for 4 °C storage of 48 and 96 h (63% and 62%, respectively). SPR_h_ were higher than SPR_k_ suggesting that minor SP (e.g., bovine serum albumin, immunoglobulins, lactoferrin) and/or proteose peptones had a low transmission rate. For T-SMilk, SPR_h_ were similar for 4 °C storage of 48 and 96 h (59% and 60%, respectively). For P2-SMilk (“no bacterial growth”), SPR_h_ were similar for 4 °C storage of 48 and 96 h (63% and 61%, respectively) and were similar to those observed for the lower heat treatment (M-SMilk). For P1-SMilk (“standard bacterial growth”), SPR_h_ were lower than those observed for P2-SMilk and decreased greatly from 48 to 96 h of 4 °C storage: 57% and 49%, respectively.

## 4. Discussion

In the dairy industry, storage at 4 °C and treatment to reduce the bacteria count are mandatory steps performed before MF. Storage of raw milk, which varies from 48–96 h depending on the collection frequency, is traditionally performed at 4 °C to limit bacterial growth before further processing. Skimmed milk is then processed, using a variety of methods, to remove, inactivate or lyse bacteria. Bacteria can be removed from milk by physical methods (e.g., bactofugation, or more effectively by 1.4 µm microfiltration) or can be inactivated and/or lysed by heat treatment, leading to denaturation of SP to a greater or lesser extent. This study provides elements to understand the individual or cumulative effects of these pre-processing steps (i.e. cold storage and microbiological stabilization) on performances (i.e., TMP, and transmission and recovery of SP over time) of skimmed milk 0.1 µm MF at 50 °C. Since the MF experiments followed the same protocol, MF performances would have been related directly to the quality of treated skimmed milk entering the MF unit. Moreover, since all milk was microfiltered in a feed-and-bleed mode of operation for 330 min, the study’s results represent industrial MF.

In this study, the effect of milk pre-processing steps was expected to be related to the presence of denatured SP, viable bacteria and/or bacterial fragments that could influence, individually or cumulatively, performances of skimmed milk MF. Other components (e.g., minerals, residual fat, spore-forming bacteria) were also considered initially but were shown to have little influence on MF performances. Concerning the mineral fraction, Svanborg et al. (2014) [12] assumed that calcium phosphorus precipitate formed during pasteurization of skimmed milk was responsible for decreasing MF performances. In our study, no precipitation was detected in heat-treated milk samples, but overnight 4 °C storage of skimmed milk after microbiological stabilization could have helped to restore the mineral equilibrium modified by heat treatment [25,26]. Since milks entering the MF unit had the same mineral equilibria, the mineral fraction in treated skimmed milk was assumed to influence MF performances little in our study. Residual fat (<2 g·kg^−1^) in skimmed milk was also shown to have little influence on MF performances or the characteristics of milk fractions. These results can be explained by observations of Le Berre and Daufin (1998) [27]. They highlighted that under given hydrodynamic conditions, fat globules with a diameter (3.5 µm) larger than the critical diameter of deposition (0.5–3.0 µm) do not occur in the fouling layer and thus, even for semi-skimmed milk, fat was not responsible for fouling during 0.1 µm MF. Consequently, these residual fat globules do not decrease MF performances. Our study also suggested that somatic cells and spore-forming bacteria do not influence strongly MF performances or the composition of permeate and retentate. Bactofugation, widely used in the industry along with heat treatment (mainly thermization) to remove spore-forming bacteria, did not decrease fouling during MF. This result may need further study to be confirmed, since only one experiment with bactofugation was performed.

**Figure 4:**
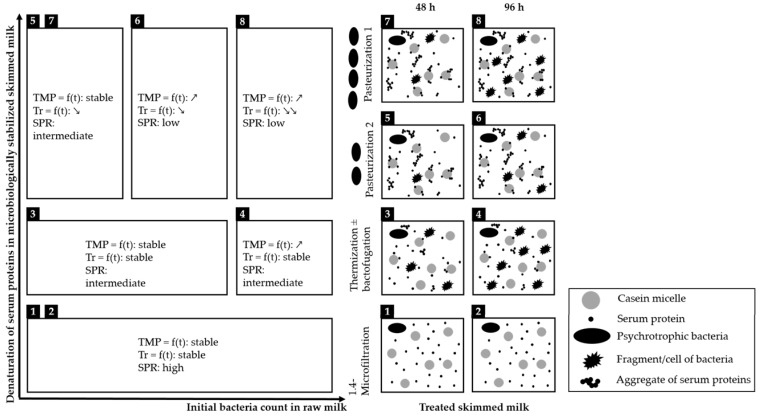
Summary of microfiltration (MF) performances as a function of initial bacteria count in raw milk and the denaturation of serum proteins in microbiologically stabilized skimmed milk. Numbers from 1 to 8 are related to the variability in composition of skimmed milk subjected to different 4 °C storage durations (48 or 96 h) and different microbiological stabilization methods (pasteurization (78 °C for 52 s); thermization (68 °C for 20 or 52 s ± bactofugation); and 1.4-µm-microfiltration (50 °C)); Abbreviations: TMP, transmembrane pressure; Tr SP, transmission of serum proteins; SPR, recovery ratio of serum protein.

Besides these initial conclusions, the key point of this study is that differences in MF performances observed when varying the history of milk (cold storage and microbiological stabilization method) could be attributed to the presence, in the skimmed milks to be filtered, of viable bacteria and/or bacterial fragments and denatured SP. Figure 4 summarizes the impact of both the initial bacteria count in raw milk (*X*-axis) and the content of denatured serum proteins (*Y*-Axis) induced by microbiological stabilization methods on MF performances of skimmed milk (evolution of transmembrane pressure, transmission of serum proteins over the time of MF and recovery of serum proteins in average permeate).

The content of residual bacteria and/or bacterial fragments influences MF performances and the characteristics of milk fractions. Since these entities are retained and concentrated in the retentate during MF, they can be assumed to form part of the fouling layer. Viable bacteria and fragments in retentate depend on both the initial bacteria count in raw milk (Figure 4) and the method used to reduce/remove the bacteria from milk. When residual bacteria and bacterial fragments are nearly absent in the skimmed milk, because they were removed (e.g., 1.4 μm -microfiltration, cases 1 and 2, Figure 4) [23,28,29,30] or because the initial bacteria count in the raw milk was low (cases 3, 5 and 7, Figure 4), hydraulic performances and transmission of SP into the permeate remain stable during MF. Conversely, when skimmed milk contains a high content of these entities (cases 4, 6 and 8, Figure 4), hydraulic performances of MF decrease, and transmission of SP into the permeate is low. This situation is observed only when skimmed milk is heat-treated, because in the absence of physical bacteria removal, milk entering the MF unit contains bacteria, either viable or lysed, and bacterial fragments resulting from the heat treatment. The bacteria count, especially that of psychrotrophic bacteria, depends on the duration of cold storage of raw milk [31]. The longer the storage duration of raw milk, the higher its bacteria count, and thus the higher the residual bacteria and cellular debris in the subsequent heat-treated milk. The higher content of these entities in raw milk cold-stored for 96 h might thus be responsible for the lower hydraulic performances of MF.

Denaturation of SP also has a notable effect on MF performances (hydraulics and characteristics of milk fractions) (Figure 4). When 1.4-µm-microfiltration or thermization that causes no or a low percentage of denaturation of SP are performed (cases 1, 2, 3 and 4, Figure 4), SP transmission remains stable during MF. Conversely, when skimmed milk entering the MF unit contains a high percentage of denatured SP (cases 5, 6, 7 and 8, Figure 4), SP transmission decreases during MF. Consequently, permeates resulting from high-heat treatment contain less SP than permeates resulting from no- or low-heat treatment, which is consistent with results of Svanborg et al. (2014) [12]. It is well known that denaturation of SP depends on the temperature and duration of the heat treatment applied [32,33,34,35,36]. The higher the intensity of heat treatment, the greater the denaturation of SP. Denaturation of β-LG is the highest during pasteurization [37,38]. The β-LG associates with κ-casein located on the surface of casein micelles, which creates β-LG-κ-CN complexes that remain at the micelle surface or form soluble particles of 30–100 nm [39]. Modification of the surface of casein micelles and/or the formation of soluble aggregates could be responsible for the decrease in MF performances observed in this study. Soluble aggregates of SP are expected to interact with components of the fouling layer and decrease MF performances, and it is assumed that SP aggregated to casein micelles would not be able to pass through the membrane. The rate of denaturation of SP and/or the nature of the aggregates formed during high-heat treatment could thus modulate the selectivity of the fouling layer formed during MF. In this way, aggregation of SP caused by heat treatment could modify the composition of milk fractions collected during MF and the compositions of the average fractions obtained at the end of the run.

This study also shows that the negative effect of SP denaturation seems to be cumulative with that of residual bacteria and/or bacterial fragments in skimmed milk. When skimmed milk contains a high content of residual bacteria and bacterial fragments and a high percentage of denatured SP (case 8, Figure 4), both the hydraulic performances and SP transmission decrease drastically. When skimmed milk contains a high content of residual bacteria and bacterial fragments but a low percentage of denatured SP (case 4, Figure 4), the hydraulic performances decrease, but the SP transmission remains stable. When skimmed milk contains a low content of residual bacteria and bacterial fragments but a high percentage of denatured SP (cases 5, 6 and 7, Figure 4), hydraulic performances and SP transmission decrease only slightly.

Consequently, we argue that the presence of residual bacteria and/or bacterial fragments decreases mainly the hydraulic performances of MF and that the presence of denatured SP influences essentially the transmission of SP. The presence of residual bacteria and bacterial fragments can also exacerbate the decrease in transmission caused by denaturation of SP. Denaturation of SP due to heat treatment may cause aggregates, which can increase membrane fouling. The presence of more protein aggregates in pasteurized milks (P1-SMilk and P2-SMilk) explains the sharper decrease in SP transmission compared to that observed for thermized milks (T-SMilk). The difference in bacteria count could explain why transmission did not change in the same way during MF of P1-SMilk and P2-SMilk from raw milk 4 °C stored for 96 h. Indeed, the initial bacteria count of raw milk 4 °C stored for 96 h used to produce P1-SMilk was higher than that used to produce P2-SMilk. The high content of bacterial fragments in milk entering the MF unit could thus exacerbate the decrease in SP transmission for pasteurized milks with more denatured SP. Further analysis is necessary to confirm these hypotheses.

## 5. Conclusions

Pre-processing steps applied to milk (cold storage and microbiological stabilization) influence the permeability and selectivity of MF. In this study, their effects were studied during MF under conditions that mimicked industrial conditions (feed-and-bleed mode). Skimmed milks microbiologically stabilized by 1.4 μm-microfiltration and obtained from raw milks cold-stored for 48 or 96 h experienced no increase in TMP and no decrease in SP transmission during MF (0.1 μm ceramic membrane used to separate milk proteins). For heat-treated skimmed milks, long-duration 4 °C storage increased TMP during MF. For low-heat-treated (thermization) skimmed milks, SP transmission was stable during MF. Conversely, for high-heat-treated (pasteurization) skimmed milks, SP transmission tended to decrease during MF, and this decrease increased as the duration of cold storage of raw milk increased.

Decreases in MF performances were attributed to the presence of bacteria (residual bacteria and bacterial fragments) and to modifications of SP (e.g., denaturation and aggregation), both of which influenced properties of the fouling layer. For skimmed milks with no or a low content of bacterial fragments (bacteria removed by 1.4-µm-microfiltration or a low TVC in raw milk) and low SP denaturation, TMP and SP transmission remained stable. Conversely, for skimmed milks with a high content of bacterial fragments (high TVC in raw milk either initially or after long-duration 4 °C storage) and low SP denaturation, TMP increased due to the fouling effects of fragments and cells, but SP transmission remained stable for 330 min of MF. For skimmed milks with both a high content of bacterial fragments and high SP denaturation, TMP increased and SP transmission decreased during MF. Negative effects caused by the presence of bacteria and denatured SP were thus cumulative.

Consequently, short-duration storage of raw milk at 4 °C is required to avoid bacterial growth and ensure good MF performances. Nonetheless, MF performances are acceptable with skimmed milk obtained from raw milk 4 °C stored for 96 h, provided the microbiological stabilization method minimizes denaturation of SP. Under these conditions, MF produces milk fractions of good quality.

## Figures and Tables

**Table 1:** Mean composition of raw milks after 48, 72 or 96 h of storage at 4 °C.

-	48 h	72 h	96 h
pH	6.71 ± 0.03 ^a^	6.80	6.69 ± 0.07 ^a^
Dornic grade	15.3 ± 0.7 ^a^	14.1	16.1 ± 0.5 ^a^
Fat	41.4 ± 2.2 ^a^	43.5	41.3± 2.3 ^b^
Ash	7.3 ± 0.0 ^b^	7.2	7.3 ± 0.2 ^b^
DM	124 ± 8 ^a^	129	127 ± 2 ^a^
TN	32.6 ± 0.5 ^a^	32.1	32.8 ± 0.5 ^a^
NCN	7.2 ± 0.2 ^a^	7.8	7.5 ± 0.2 ^a^
NPN	1.6 ± 0.1 ^a^	1.6	1.6 ± 0.1 ^a^
α-LA	0.97 ± 0.04 ^a^	1.07	0.98 ± 0.05 ^a^
β-LG	3.20 ± 0.17 ^a^	3.45	3.23 ± 0.14 ^a^

Dornic grades are in °D; fat, ash, DM (dry matter), TN (total nitrogen), NCN (non-casein nitrogen) and NPN (non-protein nitrogen) are in g·kg^−1^; contents of α-LA (α-lactalbumin) and β-LG (β-lactoglobulin) are in g·L^−1^, ^a^—*n* = 4, ^b^—*n* = 3.

**Table 2:** Total viable count (TVC) and psychrotrophic bacteria count (PBC) (CFU·mL^−1^) of raw milks after 48, 72 or 96 h of storage at 4 °C, before application of a microbiological stabilization method: 1.4 μm microfiltration (M-SMilk), thermization (T-SMilk) or pasteurization (P1-SMilk, P2-SMilk).

Raw Milk Used to Produce	Storage	TVC	PBC
M-SMilk	48 h	9.7 × 10^4^	4.7 × 10^2^
	96 h	2.7 × 10^5^	2.3 × 10^4^
T-SMilk	48 h	7.2 × 10^3^	1.3 × 10^2^
	72 h	1.6 × 10^4^	1.7 × 10^2^
	96 h	3.3 × 10^4^	2.2 × 10^2^
P1-SMilk	48 h	2.3 × 10^4^	<10^2^
	96 h	>5 × 10^4^	>10^4^
P2-SMilk	48 h	1.5 × 10^4^	<10
	96 h	1.4 × 10^4^	<10

**Table 3:** Composition of skimmed milks subjected to a microbiological stabilization method: 1.4 μm microfiltration (M-SMilk), thermization (T-SMilk) or pasteurization (P1-SMilk, P2-SMilk). Storage duration at 4 °C (48, 72 or 96 h) is for raw milk before skimming and microbiological stabilization.

-	M-SMilk		T-SMilk			P1-SMilk		P2-SMilk	
	48 h	96 h	48 h	72 h	96 h	48 h	96 h	48 h	96 h
pH	6.72	6.70	6.79	6.57	6.64	6.73	6.71	6.75	6.71
Dornic grade	15.3	15.4	14.8	15.9	15.0	15.0	15.3	16.0	16.8
Fat	0.5	0.5	0.5	0.5	0.5	2.0	0.5	ND	ND
Ash	7.6	7.7	7.5	7.4	8.9	7.6	7.7	ND	ND
Total calcium	ND	ND	1293	1292	1305	1220	1238	ND	ND
Soluble calcium	ND	ND	332	327	330	315	310	ND	ND
Phosphate	ND	ND	882	871	881	821	838	ND	ND
Citrate	ND	ND	1539	1530	1516	1460	1491	ND	ND
DM	93	93	94	93	94	93	92	92	92
TN	34.0	34.8	33.7	33.6	33.6	33.7	34.4	33.6	33.4
NCN	8.3	8.4	7.7	8.0	8.1	7.5	7.8	6.7	7.1
NPN	1.7	1.7	1.6	1.6	1.6	1.5	1.7	1.8	1.6
α-LA	1.06	1.04	1.01	0.99	1.15	1.04	1.05	0.95	0.99
β-LG	3.41	3.39	3.46	3.40	3.43	3.26	3.26	2.88	2.95
DR	< 1	< 1	2	5	4	9	13	11	8
TVC	6.7 × 10^2^	6.0 × 10^2^	2.2 × 10^4^	2.5 × 10^4^	3.2 × 10^4^	>3 × 10^2^	>3 × 10^2^	4.5 × 10^2^	3.5 × 10^2^
PBC	<10	10	<10	<10	<10	<10	<10	ND	ND

Dornic grades are in °D; fat and ash contents are in g·kg^−1^; total calcium, soluble calcium, phosphate and citrate contents are in mg·kg^−1^; DM (dry matter), TN (total nitrogen), NCN (non-casein nitrogen), NPN (non-protein nitrogen) contents are in g·kg^−1^; α-LA (α-lactalbumin) and β-LG (β-lactoglobulin) contents are in g·L^−1^; DR (serum protein denaturation) is in %; TVC (total viable count) and PBC (psychrotrophic bacteria count) are in CFU·mL^−1^. Abbreviations: ND, not determined.

**Table 4:** Transmembrane pressure (TMP)_(±1 experimental error value)_(10^5^ Pa) at the beginning of MF (TMP_i_), at the end of MF (TMP_f,_) and change in TMP (ΔTMP) after 330 min microfiltration for 1.4 μm microfiltered (M-SMilk), thermized (T-SMilk) or pasteurized (P1-SMilk, P2-SMilk) skimmed milks. Storage time at 4 °C (48 or 96 h) is for raw milk before skimming and microbiological stabilization. Operating conditions: 0.1 μm ceramic membrane; uniform transmembrane pressure system; permeation flux, Jp = 75 L·h^−1^·m^−2^; volume reduction ratio = 3.0; T = 50 °C).

-	48 h Storage of Raw Milk			96 h Storage of Raw Milk		
Milk Type	TMP_i_	TMP_f_	ΔTMP	TMP_i_	TMP_f_	ΔTMP
M-SMilk	0.53 ± 0.04	0.56 ± 0.04	0.03 ± 0.08	0.50 ± 0.04	0.53 ± 0.04	0.03 ± 0.08
T-SMilk	0.52 ± 0.04	0.56 ± 0.04	0.04 ± 0.08	0.56 ± 0.04	0.69 ± 0.04	0.13 ± 0.08
P1-SMilk	0.52 ± 0.04	0.55 ± 0.04	0.03 ± 0.08	0.57 ± 0.04	0.71 ± 0.04	0.14 ± 0.08
P2-SMilk	0.48 ± 0.04	0.52 ± 0.04	0.04 ± 0.08	0.47 ± 0.04	0.54 ± 0.04	0.07 ± 0.08

For M-SMilk, ∆TMP remained low (0.03 × 10^5^ Pa), regardless of the 4 °C storage duration of raw milk (Table 4). After 4 °C storage for 48 h, heat-treated milks (T-SMilk, P1-SMilk and P2-SMilk) also had low ∆TMP (0.04 × 10^5^ Pa). For T-SMilk, bactofugation had little influence on ∆TMP (results not shown). For pasteurized milks, ∆TMP were similar (0.04 × 10^5^ Pa) for P1-SMilk (low bacteria count, 2.0 g·kg^−1^ of residual fat) and P2-SMilk (low bacteria count, 0.5 g·kg^−1^ of residual fat). Conversely, after 4 °C storage for 96 h, TMP increased for heat-treated milks: by 23% for T-SMilk (by 15% when stored for 72 h; results not shown), and by 25% and 15% for P1-SMilk and P2-SMilk, respectively.

**Table 5:** Recovery ratios of serum proteins (SP) and content of SP in average permeate obtained after microfiltration of microbiologically stabilized skimmed milks: 1.4 μm microfiltration (M-SMilk), thermization (T-SMilk) or pasteurization (P1-SMilk, P2-SMilk) after 48 h or 96 h of 4 °C storage. Operating conditions: 0.1 μm ceramic membrane; uniform transmembrane pressure system; permeation flux, Jp = 75 L·h^−1^·m^−2^; volume reduction ratio = 3.0; T = 50 °C).

-	M-SMilk		T-SMilk		P1-SMilk		P2-SMilk	
	48 h	96 h	48 h	96 h	48 h	96 h	48 h	96 h
SPR_k_	56	54	50	55	48	41	50	51
SPR_h_	63	62	59	60	57	49	63	61
C_Ap,k_	5.15	5.39	4.54	4.99	4.41	3.78	4.21	4.57
C_Ap,h_	4.13	4.10	3.91	3.95	3.64	3.13	3.64	3.60

SPR_k_ (recovery ratio of total SP) and SPR_h_ (recovery ratio of SP present in the aqueous phase and able to pass the membrane) in %; C_Ap,k_ (total SP content in average permeate) in g·kg^−1^; C_Ap,h_ (sum of α-LA and β-LG content in average permeate) in g·L^−1^.
